# Supplementary material for: Electrocatalytic Oxygen Reduction Reaction of Graphene Oxide and Metal-Free Graphene in an Alkaline Medium
Source: Nanomaterials (Basel). 2023 Apr 8;13(8):1315. doi: 10.3390/nano13081315 (PMC10146927; doi:10.3390/nano13081315)
Supplement: Supplementary file 1 [file nanomaterials-13-01315-s001.zip › nanomaterials-2311438-supplementary.pdf]

Supplementary Materials

Electrocatalytic Oxygen Reduction Reaction of Graphene Oxide and Metal-Free Graphene in an Alkaline Medium

Saravanan Nagappan [1,*], Malarkodi Duraivel [2], SeongHoon Han [3], Mohammad Yusuf [1], Manjiri Mahadadalkar [1], KyeongMun Park [1], Amarajothi Dhakshinamoorthy [4], Kandasamy Prabakar [2], Sungkyun Park [3], Chang-Sik Ha [5], Jae-Myung Lee [6] and Kang Hyun Park [1,*]

[1] Department of Chemistry, Chemistry Institute for Functional Materials, Pusan National University, 2 Busandaehak-ro 63beon-gil, Geumjeong-Gu, Busan 46241, Republic of Korea; yusuf.chem.kor@gmail.com (M.Y.); manjeeri@gmail.com (M.M.); pkmkm951@naver.com (K.P.)
[2] Department of Electrical Engineering, Pusan National University, 2 Busandaehak-ro 63beon-gil, Geumjeong-Gu, Busan 46241, Republic of Korea; malarshalu94@gmail.com (M.D.); prabakar@pusan.ac.kr (K.P.)
[3] Department of Physics, Pusan National University, 2 Busandaehak-ro 63beon-gil, Geumjeong-Gu, Busan 46241, Republic of Korea; han1411@pusan.ac.kr (S.H.); psk@pusan.ac.kr (S.P.)
[4] School of Chemistry, Madurai Kamaraj University, Palkalai Nagar, Madurai 625021, Tamil Nadu, India; admguru@gmail.com (A.D.)
[5] Department of Polymer Science and Engineering, Pusan National University, 2 Busandaehak-ro 63beon-gil, Geumjeong-Gu, Busan 46241, Republic of Korea; csha@pusan.ac.kr (C.-S.H.)
[6] Department of Naval Architecture and Ocean Engineering, Pusan National University, Busan 46241, Republic of Korea; jaemlee@pusan.ac.kr (J-M.L.)
* Correspondence: saravananagappan@gmail.com (S.N.); chemistry@pusan.ac.kr (K.H.P.)

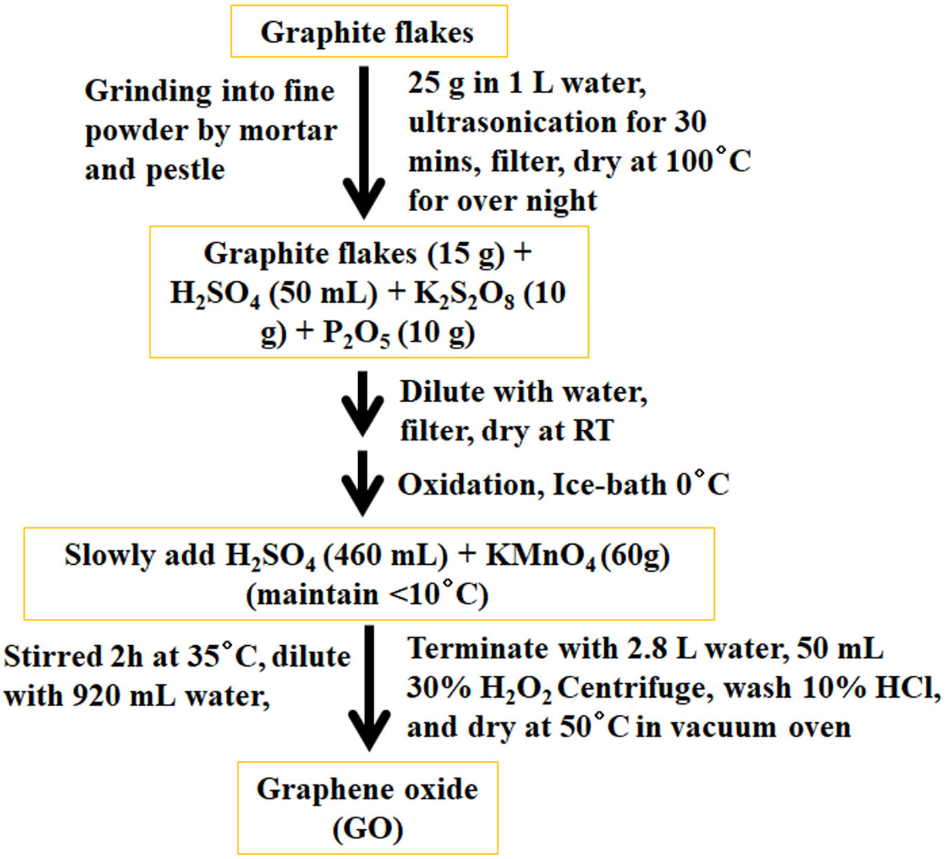

Figure S1. Flow chart for the synthesis of GO.

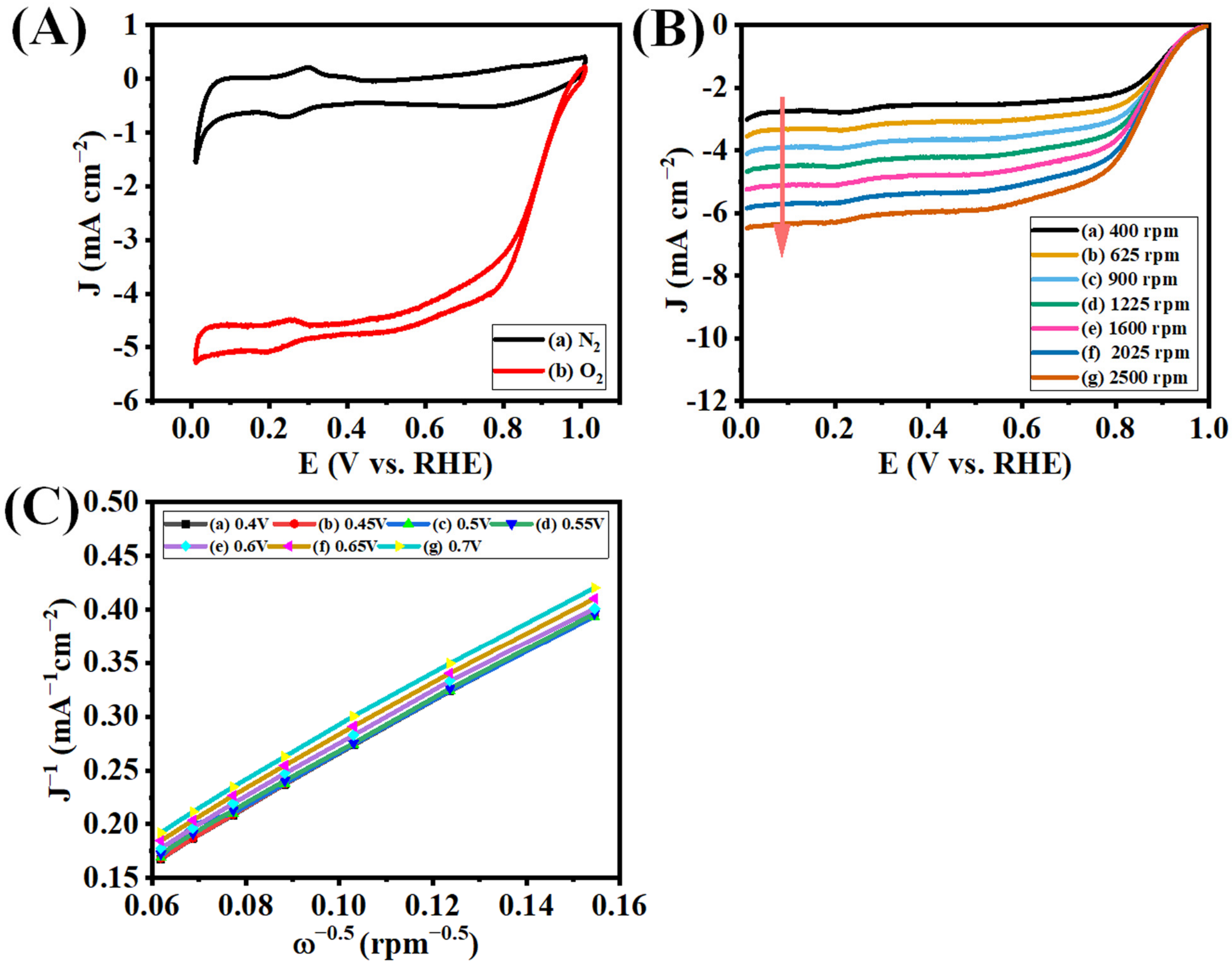

Figure S2. (A) CV graphs of Pt/C in N_2 and O_2 saturated aqueous 0.1M KOH electrolytes. (B and C) LSV at different rpm and K-L plots of Pt/C in O_2 saturated aqueous 0.1M KOH electrolyte at the rotation speed of 1600 rpm. All the experiments were performed at the scan rate of 10 mV s^{-1} at room temperature.

Table S1. Comparison study of graphene-based electrocatalyst for ORR.

Catalyst	Electrolyte (KOH)	ORR onset potential (E_{onset}) (V vs. RHE)	ORR $E_{1/2}$ (V vs. RHE)	Electron transfer number (n)	Ref.
GO	0.1 M	0.8	0.66	1.69	This Work
G100-1B	0.1 M	0.84	0.77	3.76	This Work
G100-2B	0.1 M	0.84	0.74	3.41	This Work
N-GRW	0.1 M	0.92	0.84	3.95	[49]
N-Graphene/CNT	0.1 M	0.88	0.7	3.22	[50]
Fe3Co@NG-C	0.1 M	1.03	0.86	3.90	[51]
N*g*-C@G	0.1 M	0.96	0.81	3.96	[16]
N-pGF	0.1 M	0.86	0.76	3.9	[52]
N-GA-150	0.1 M	0.65	0.78	3.7	[53]
N-GDY	0.1 M	0.65	0.78	3.7	[54]
NGM	0.1 M	0.89	0.77	3.8	[55]
NPGC-950	-	0.91	0.78	3.79	[56]

References

16. Begum, H.; Ahmed, M.S.; Kim, Y.B. Nitrogen-Rich Graphitic-Carbon@graphene as a Metal-Free Electrocatalyst for Oxygen Reduction Reaction. *Sci. Rep.* **2020**, *10*, 12431.
49. Yang, H. Bin; Miao, J.; Hung, S.-F.; Chen, J.; Tao, H.B.; Wang, X.; Zhang, L.; Chen, R.; Gao, J.; Chen, H.M.; et al. Identification of Catalytic Sites for Oxygen Reduction and Oxygen Evolution in N-Doped Graphene Materials: Development of Highly Efficient Metal-Free Bifunctional Electrocatalyst. *Sci. Adv.* **2016**, *2*, e1501122.
50. Tian, G.-L.; Zhao, M.-Q.; Yu, D.; Kong, X.-Y.; Huang, J.-Q.; Zhang, Q.; Wei, F. Nitrogen-Doped Graphene/Carbon Nanotube Hybrids: In Situ Formation on Bifunctional Catalysts and Their Superior Electrocatalytic Activity for Oxygen Evolution/Reduction Reaction. *Small* **2014**, *10*, 2251–2259.
51. Li, J.; Liu, J.-X.; Gao, X.; Goldsmith, B.R.; Cong, Y.; Zhai, Z.; Miao, S.; Jiang, Q.; Dou, Y.; Wang, J.; et al. Nitrogen-Doped Graphene Layers for Electrochemical Oxygen Reduction Reaction Boosted by Lattice Strain. *J. Catal.* **2019**, *378*, 113–120.
52. Bang, G.S.; Shim, G.W.; Shin, G.H.; Jung, D.Y.; Park, H.; Hong, W.G.; Choi, J.; Lee, J.; Choi, S.-Y. Pyridinic-N-Doped Graphene Paper from Perforated Graphene Oxide for Efficient Oxygen Reduction. *ACS Omega* **2018**, *3*, 5522–5530.
53. Xue, Q.; Ding, Y.; Xue, Y.; Li, F.; Chen, P.; Chen, Y. 3D Nitrogen-Doped Graphene Aerogels as Efficient Electrocatalyst for the Oxygen Reduction Reaction. *Carbon N. Y.* **2018**, *139*, 137–144.
54. Lv, Q.; Si, W.; Yang, Z.; Wang, N.; Tu, Z.; Yi, Y.; Huang, C.; Jiang, L.; Zhang, M.; He, J.; et al. Nitrogen-Doped Porous Graphdiyne: A Highly Efficient Metal-Free Electrocatalyst for Oxygen Reduction Reaction. *ACS Appl. Mater. Interfaces* **2017**, *9*, 29744–29752.
55. Tang, C.; Wang, H.-F.; Chen, X.; Li, B.-Q.; Hou, T.-Z.; Zhang, B.; Zhang, Q.; Titirici, M.-M.; Wei, F. Topological Defects in Metal-Free Nanocarbon for Oxygen Electrocatalysis. *Adv. Mater.* **2016**, *28*, 6845–6851.
56. Men, B.; Sun, Y.; Li, M.; Hu, C.; Zhang, M.; Wang, L.; Tang, Y.; Chen, Y.; Wan, P.; Pan, J. Hierarchical Metal-Free Nitrogen-Doped Porous Graphene/Carbon Composites as an Efficient Oxygen Reduction Reaction Catalyst. *ACS Appl. Mater. Interfaces* **2016**, *8*, 1415–1423.
